# Mental health literacy of resettled Iraqi refugees in Australia: knowledge about posttraumatic stress disorder and beliefs about helpfulness of interventions

**DOI:** 10.1186/s12888-014-0320-x

**Published:** 2014-11-18

**Authors:** Shameran Slewa-Younan, Jonathan Mond, Elise Bussion, Yaser Mohammad, Maria Gabriela Uribe Guajardo, Mitchell Smith, Diana Milosevic, Sanja Lujic, Anthony Francis Jorm

**Affiliations:** Mental Health, School of Medicine, University of Western Sydney, Locked Bag 1797, Penrith South DC, Sydney, NSW Australia; Department of Psychology, Macquarie University, Sydney, NSW Australia; New South Wales Refugee Health Service, Liverpool, NSW Australia; South Western Sydney Local Health District Eastern Campus, Liverpool Hospital, Liverpool, NSW Australia; Centre for Health Research, School of Medicine, The University of Western Sydney, Sydney, NSW Australia; Centre for Mental Health, Melbourne School of Population and Global Health, University of Melbourne, Melbourne, Vic Australia

**Keywords:** Mental health literacy, Posttraumatic stress disorder, Iraqi refugees, Help-seeking, attitudes

## Abstract

**Background:**

Resettled refugees are a particularly vulnerable group. They have very high levels of mental health problems, in particular, trauma-related disorders, but very low uptake of mental health care. Evidence suggests that poor “mental health literacy”, namely, poor knowledge and understanding of the nature and treatment of mental health problems is a major factor in low or inappropriate treatment-seeking among individuals with mental health problems. This study used a culturally adapted Mental Health Literacy Survey method to determine knowledge of, and beliefs about, helpfulness of treatment interventions and providers for posttraumatic stress disorder (PTSD) amongst resettled Iraqi refugees.

**Methods:**

225 resettled Iraqi refugees in Western Sydney attending the Adult Migrant English Program (AMEP), federally funded English language tuition, were surveyed. A vignette of a fictional character meeting diagnostic criteria for PTSD was presented followed by the Mental Health Literacy Survey. PTSD symptomology was measured using the Harvard Trauma Questionnaire part IV (HTQ part IV), with Kessler Psychological Distress Scale (K10) used to measure levels of general psychological distress.

**Results:**

Only 14.2% of participants labelled the problem as PTSD, with “a problem with fear” being the modal response (41.8%). A total of 84.9% respondents indicated that seeing a psychiatrist would be helpful, followed by reading the Koran or Bible selected by 79.2% of those surveyed. There was some variation in problem recognition and helpfulness of treatment, most notably influenced by the length of resettlement in Australia of the respondents.

**Conclusions:**

These findings have important implications for the design and implementation of mental health promotion and treatment programs for resettled refugees and those who work with them.

**Electronic supplementary material:**

The online version of this article (doi:10.1186/s12888-014-0320-x) contains supplementary material, which is available to authorized users.

## Background

Refugee mental health refers to the specific psychological health concerns of those who have been forcibly displaced from their country of origin, due to circumstances defined by the 1951 Refugee Convention [1]. Some 108, 000 refugees were resettled in Australia in the years between 2001 and 2010 [2], from a diverse range of nationalities and cultural backgrounds. To be granted refugee status is to have suffered persecution on the basis of race, religion, nationality, social group or political opinion, or to have had a genuine and justified fear of the same [1]. This persecution may include, and is not limited to, instances of torture [3], deprivation of necessities such as adequate food and clean water and experiences of civil war [4]. Throughout this period, refugees are unlikely to have had uninterrupted access to adequate primary healthcare [5]. Following resettlement, refugees may experience feelings of grief due to separation from their culture, family and existing social supports, and language barriers can further contribute to feelings of isolation [6]. Consequently, refugees are a population particularly vulnerable to disorders of both physical [7] and mental health [8,9], even after resettlement.

A significant challenge in refugee mental health is willingness to access care. Importantly, resettled refugees are significantly less likely than the general population to utilise hospital services [10], despite a higher prevalence of trauma related mental health disorders. Indeed, one meta-analysis found that refugees resettled in Western nations may be up to ten times more likely to suffer from posttraumatic stress disorder (PTSD) than their age matched controls in the general population [8]. Another systematic review reported weighted prevalence rates across the surveys of 30.6 and 30.8% for PTSD and depression, respectively [11]. In a Swiss study of migrants originating from Bosnia and Turkey, it was noted that when medical attention is sought, somatic symptoms dominated the initial presentation. While psychological distress was revealed to be a more common disturbance than somatic problems, this was only reported as a result of focused questioning [12]. A lack of understanding of what constitutes mental illness may present a barrier to accessing care, due in part to differences in cultural perceptions of mental health [13]. As such, gaining an appreciation of an individual’s understanding of mental health can provide insight into their willingness to access care for mental health concerns.

Mental health literacy (MHL) refers to, “knowledge and beliefs about mental disorders which aid their recognition, management or prevention” [14]. This encompasses (a) the ability to recognize specific disorders; (b) knowledge of how to seek mental health information; (c) knowledge of risk factors and causes, of self-treatments, and of professional help available; and (d) attitudes that promote recognition and appropriate help-seeking [14]. Increasing MHL can achieve dual goals. First, to empower the public with an understanding of mental disorders, thereby facilitating prevention, early intervention and treatment within the community [15]. Second, to empower individuals with the means by which to make an informed decision about accessing mental health care.

While multiple national surveys of MHL [16] have ascertained the general attitudes of those living in Australia, little is known about the mental health literacy of resettled refugees. As a diverse, heterogeneous group, originating from a range of cultures, nationalities and religions, studies of refugee health are best undertaken specific to a refugee subgroup. In Australia, Iraqi refugees are one particularly prominent subgroup, with 4064 offshore visas granted to Iraqi-born individuals in financial year 2012-2013 alone. This was the primary country of origin for offshore visas in this time period, comprising 32.47% of the total visas granted [17]. Consequently, they are a group of particular relevance to Australian health care professionals, and are the population surveyed in this study.

A recent study compared the mental health beliefs and knowledge of Sydney-based Iraqi and Sudanese refugees to those of their Australian-born counterparts [18]. Participants (n = 64) were given vignettes depicting characters with PTSD and major depressive disorder, for which they were directed to identify a diagnosis and appropriate treatment approaches. Refugees of both Iraqi and Sudanese origin demonstrated significantly lower use of correct psychiatric labels, and a limited knowledge of Western treatment approaches of mental disorders. Although these findings suggest that resettled refugees have low mental health literacy when judged against the concepts of Western mental health professionals, the generalizability of the findings was limited by the use of vignettes written from a Western perspective, the recruitment of participants from a treatment-seeking sample, and the comparatively small sample size.

The goal of the current study was to add to this limited evidence base by examining the mental health literacy of resettled refugees in Australia. Specifically, this study sought to determine knowledge of, and beliefs about, helpfulness of treatment interventions and providers for PTSD amongst resettled Iraqi refugees resettled in Australia. We sought to improve upon May et al’s [18] study by recruiting a larger sample of participants from the general population and by using a culturally adapted mental health literacy survey methodology.

## Methods

### Study design and participants

All participants were resettled Iraqi refugees attending the Adult Migrant English Program (AMEP) at several different colleges in the Western Sydney region of Australia. AMEP is a federally funded English language tuition program that is offered to eligible migrants and humanitarian entrants to Australia who do not have functional English. Participation entailed the completion of a pen-and-paper, respondent-based survey, conducted on the college campuses by means of in-person interviews. Bilingual investigators attended the colleges between March and November 2013 and informed potential participants of the project through the distribution of flyers. The flyers outlined the aims and nature of the research and indicated that all participants would receive a food gift voucher in the amount of AUD $25.00 upon completion of the survey in appreciation of their time. Those interested were asked to notify their language tutors, who would in turn, contact the investigators on their behalf. Written consent was obtained from all participants, following a more detailed description of the survey content, prior to commencement of each interview. Inclusion criteria were having been born in Iraq, having left Iraq no earlier than 1991, being fluent in Arabic and/or English, and being between the ages of 18 and 70 years. Approval for the study was obtained from the University of Western Sydney Human Research Ethics Committee. Interviews, administering the instruments described below, in addition to the collection of demographic information, were conducted by two bilingual (fluent in Arabic and English) members of the research team and extended up to 90 minutes. Additionally, all participants were provided with translated information sheets containing details of local specific mental health services available.

### Measures

#### The Mental Health Literacy Survey

The survey was modelled on Jorm et al.’s [14] protocol, with modifications by the authors (SSY, JM, YM and AFJ) for the study of PTSD in refugees. Specifically, the vignette was developed based on the consensus of several authors (SSY and YM) experienced in the assessment and clinical treatment of PTSD in Iraqi refugees. Care was taken to ensure the vignette was culturally valid and the final survey was translated into Arabic and independently back-translated into English, using the services of nationally accredited translation and interpreting service. All discrepancies were checked and rectified by the translators, and the research team [19]. The vignette describing a fictional Iraqi refugee “Miriam or Dawood” (sex of vignette character was matched to sex of participant being interviewed), who had been exposed to trauma prior to leaving Iraq and who was suffering symptoms of PTSD, according to criteria outlined in 4th edition of the Diagnostic and Statistical Manual of Mental Disorders (DSM IV- TR, American Psychiatric Association) was read aloud by the interviewer (see Additional file 1). A prompt card in Arabic was provided so that the participant could follow the description as it was read and participants were advised they could refer back to vignette at any time during the interview. Following the presentation of vignette, participants were asked: ‘What would you say is Miriam’s/Dawood’s main problem’? Participants were required to choose only one answer from a number of options. Listed in random, these were: ‘fear’; ‘no real problem’; ‘just a phase’; ‘depression’; ‘weak character’; ‘nervous breakdown’; ‘posttraumatic stress disorder’; ‘serious medical condition (e.g. brain tumour)’; ‘stress’; ‘not integrating well in Australia/homesickness’; and ‘physical condition (e.g. migraine or back pain). Participants beliefs about helpfulness of various interventions for the problem described in vignette were also assessed (Table 1). Specifically, participants were asked whether each of a number of interventions within each of two categories – treatments activities and people, would be helpful, harmful or neither (helpful nor harmful) for the person described in the vignette. Additionally, participants were asked which intervention within each category they believed would be most helpful for this person.

**Table 1:** **Perceived helpfulness of interventions for PTSD vignette (** ***n*** **= 225)**

**Interventions**	**Helpful**	**Harmful**	**Neither**	Most helpful*
*Treatments and activities*				
Reading Koran or Bible	79.2	1.8	17.3	19.0
Finding new hobbies	75.2	2.7	19.0	8.0
Psychotherapy focusing on relationships with others	73.0	3.5	20.8	5.5
Prayer session	66.8	3.1	28.8	4.4
Improving diet or exercise	66.8	2.7	26.5	2.7
Psychotherapy focusing on past	64.2	10.2	23.0	9.3
Relaxation	62.4	4.4	30.1	2.2
Psychotherapy focusing on changing thoughts	61.5	9.3	27.4	16.4
Getting information about problem	60.2	9.3	27.4	4.9
Reading a self-help book	59.3	3.1	34.5	0.4
Trying to deal with problem alone	58.0	8.8	31.4	6.6
Talking about problem	46.0	22.1	29.2	7.5
Admission to a psychiatric hospital	29.2	34.1	33.6	4.9
Traditional therapies	13.7	37.2	46.0	0.9
Hypnosis	5.8	43.3	46.5	0.9
Drinking alcohol to relax	4.0	75.7	15.9	0.4
*People*				
Psychiatrist	84.5	4.9	8.8	35.4
Family member	66.4	3.1	28.3	11.5
GP	66.4	1.8	27.9	9.7
Psychologist	65.0	3.5	27.4	8.4
Religious leader	61.1	3.1	31.9	11.1
Close female friend	50.0	6.6	40.3	6.2
Iraqi social group/club	48.2	8.8	39.4	7.1
Close male friend	42.5	11.9	40.7	2.7
Community mental health worker	42.0	12.4	42.5	4.9
Community religious organisation	38.9	5.8	49.6	0.9
Telephone counselling line	26.1	10.6	56.2	0

*Percentage of sample rating the specific intervention item as ‘the most helpful’ for treating problem described in vignette.

#### General psychological distress

To assess the general symptoms of anxiety and depression, the Kessler Psychological Distress Scale (K10) [20] was used. The K10 is a self-report questionnaire of depression and general mental disorder. Scores range from 10 to 50, with established thresholds of low to mild (10-21), moderate (22-29) and severe distress (≥30) applied to provide a measure of symptoms among the participants. The K10 has good psychometric properties with internal consistency of α = .86 reported for Arabic speaking refugees [21]. Cronbach’s alpha in the current study was .94.

#### Trauma and PTSD symptoms

The Harvard Trauma Questionnaire (HTQ) [22] is a widely used, cross-cultural self-report checklist assessing the occurrence and frequency of various types of traumatic events as well as PTSD symptomatology. It consists of four parts: part I, which assesses the number and types of potentially traumatic events (PTE) experienced and/or witnessed, part II, an open ended description of the traumatic narrative, part III enquires about head injury, and part IV, which addresses the occurrence and severity of PTSD symptomatology. Only parts I and IV were included in the present study, being directly relevant to the assessment of PTSD. A score of 2.5 or above on Part IV is considered to indicate a high probability of clinically significant PTSD symptomatology [22]. The HTQ has very good psychometric properties, including high test-retest reliability and internal consistency for part IV [22]. Cronbach’s alpha in the current study was .93.

### Statistical Analyses

With 225 respondents, the study had 80% power at 5% significance level to detect a medium effect size of between 0.19 to 0.24 using 1 to 5 degrees of freedom Chi-square test, and an effect size of 0.38 for Mann-Whitney tests of associations between key demographic variables, symptom levels and specific aspects of MHL.

Statistical analysis was carried out using IBM SPSS Statistics version 22.0. Continuous variables are presented as median and interquartile range (IQR), whereas categorical variables were expressed as percentage (%) frequencies. The effect of socio-demographic characteristics (age, sex, length of time in Australia, religion and education) and symptom levels on the K10 and HTQ Part IV (as defined in the scale descriptions) on responses regarding problem recognition and beliefs about interventions were examined using Mann-Whitney U tests, Kruskal-Wallis tests, Spearman’s rank correlations or Chi-square tests, as appropriate (for the purpose of this analysis, responses to questions concerning the perceived helpfulness of particular interventions were recoded: helpful = 1, harmful = -1, and neither = 0). Pairwise post hoc comparisons of significant socio-demographic characteristics were performed using Dunn’s procedure [23] with a Bonferroni correction for multiple comparisons, with adjusted p-values reported. Missing data was low, in the order of 7.2% and was handled by listwise deletion. Where appropriate, data for levels of general psychological distress and PTSD symptomatology derived from the Second (2007) Australian National Survey of Mental Health and Wellbeing (NSMHWB) [24] and responses from the 2011 National Survey of Mental Health Literacy and Stigma (NSMHLS) [16] were used for comparative purposes.

## Results

Interviews were conducted with a total of 225 participants over the 10-month data collection period. Their demographic and clinical characteristics are shown in Table 2.

**Table 2:** **Demographic and clinical characteristics of participants**

**Characteristics**	**N (Total = 225)** ^**i**^	**%**
Gender		
Male	98	43.6
Female	127	56.4
Age in years, mean (SD)	37.9 (14.2)	-
Years of education, mean (SD)	10.7 (3.9)	-
Months in Australia, mean (SD)	59.1 (64.5)	-
Months externally displaced, mean (SD)	49.9 (70.4)	-
Religion		
Christian	102	45.3
Muslim	86	38.2
Mandean	37	16.4
Marital Status		
Never Married	51	22.6
Married/Partner	151	66.8
Divorced	5	2.2
Widowed	12	5.3
K10 Psychological Distress		
Low to mild	88	40.9
Moderate	42	19.5
Severe	85	39.5
Probable PTSD^ii^	70	31.1

^i^May not add to 225 due to missing data.

^ii^HTQ ≥2.5.

### Clinical characteristics

Approximately one third (31.1%) of participants met the threshold (≥2.5) for clinically significant PTSD symptomatology according to the HTQ. In comparison, reported rates of PTSD in NSMHWB ranged from 6.4% for 12 months prevalence to 12.2% for lifetime presence, which are between two to five fold less than in our sample. According to the cut-off scores for the K10 used in the NSMHWB, 39.1% of participants had severe psychological distress, 18.7% had moderate distress, and 37.8% low to mild distress. By way of comparison, 2.6% of the general Australian population experienced severe psychological distress according to the 2007 NSMHWB [24].

#### Mental Health Literacy Survey

In response to the question ‘What would you say is Miriam’s/Dawood’s main problem?’ 94 respondents (41.8%) chose fear, and a further 44 (19.6%) chose depression. Only 32 respondents (14.2%) used the correct psychiatric label of PTSD, whereas 26 (11.6%) believed it was a nervous breakdown. Collectively, these responses accounted for 87.2% of all responses. By way of comparison, 34.3% of the general Australian public surveyed in the 2011 National Survey of Mental Health Literacy and Stigma (NSMHLS) [16] gave the correct psychiatric label to a PSTD vignette.

Table 1 shows the percentage of respondents who considered interventions within each subcategory (treatments or people) as ‘helpful’, ‘harmful’ or ‘neither’, for the problem described. As can be seen, reading the Koran or Bible was the treatment most frequently indicated as being helpful by participants (80.0%). Next most commonly endorsed helpful activity was ‘finding new hobbies’ (76.0%), followed by 73.3% citing ‘psychotherapy focusing on relationships with others’. The single most helpful treatments were reading the Koran or Bible (19.1%) and ‘psychotherapy focusing on changing thoughts’ (17.3%). By comparison, the top three helpful interventions selected by participants in the NSMHLS were ‘physical activity’ (93.5%) ‘get out more’ (88%), and ‘learn relaxation’ (87.9%). Of concern is the finding that 57.8% of the Iraqi participants reported that ‘trying to deal with the problem alone’ would be helpful, which is in direct contrast to 6% of respondents in NSMHLS who believed such an action would be helpful. In terms of assistance from people, the Iraqi participants most frequently cited psychiatrists as being helpful (84.9%), followed by a family member (67.1%) and GP (66.7%). In the NSMHLS, by comparison, the persons considered most likely to be helpful were counsellors (91.3%), GPs (85.6%) and psychiatrists (84.2%).

### Factors affecting response to particular questions

Significant differences between the main problem identified and demographic factors were found only for time spent in Australia variable (H(9) = 22.61, p = .007). The post hoc analysis showed significant differences in median time spent in Australia for participants describing the problem in the vignette as “just a phase” (33.67) and those selecting “homesickness” (186.25) (p = 0.013) or “physical condition” (222.50) (p = 0.017), indicating that participants who recently arrived in Australia were more likely to describe the problem as “just a phase”.

Significant differences were also found among *most* helpful treatments and length of stay in Australia (H(16) = 31.17, p = 0.013). In particular, participants who recently arrived in Australia were significantly more likely to select “trying to deal with a problem alone” compared with “admission to psychiatric hospital” among those who had been living in Australia longer.

With regards to individual treatment helpfulness, participants who had been living in Australia longer were more likely to endorse activities such reading a self-help book (rs = .224, p = .001), reading the Bible or Koran (rs = .181, p = .007) and improving diet/exercise (rs = .169, p = .012), and less likely to endorse drinking alcohol (rs = -0.212, p = 0.002) as helpful compared with those who were more recently resettled in Australia. Those with higher levels of education (12 years or more) were more likely to believe that finding new hobbies (rs = .247, p < .001) as being helpful. Christians were more likely to consider “psychotherapy focused on changing thinking” as helpful than Mandaeans (p = .018, Mean Rank = 122.79, and 93.74 respectively), whereas Mandaeans were more likely to consider “traditional therapy” as helpful as Christians (p = 0.016, Mean Rank = 129.11 and 97.60 respectively). Participants without probable PTSD were more likely to believe that getting information about the problem would be a helpful activity (p = .012, Mean Rank = 116.14, and 96.07, respectively).

In terms of treatment providers, those participants who had been living in Australia for longer believed that GPs (rs = .208, p = .002), psychologists (rs = .231, p = .001), community mental health teams (rs = .241, p < .001) and telephone counselling (rs = .208, p = .002) were more helpful compared to those recently arrived. Muslims were more likely than Christians to believe that seeking help from “close male friend” is helpful (p = .008, Mean Rank = 121.88, and 96.24 respectively).

## Discussion

Knowledge of mental health disorders and beliefs about the helpfulness of treatments are aspects considered to be central to mental health literacy. This is the first comprehensive study of the MHL of resettled Iraqi refugees. Our results indicated that only 14.2% of the sample survey used the correct psychiatric label of PTSD to describe the problem in the vignette and that this was not influenced by participants’ socio-demographic characteristics or their symptomology levels. Most participants believed that reading a religious text such as the Bible or Koran and seeking help from a psychiatrist were the interventions most likely to be helpful for someone suffering from symptoms described in the vignette. There was some variation in beliefs regarding treatment according the length of time in Australia of the respondents and their levels of education and religious affiliations.

In the 2011 NSMHLS [16], 34.4% of the general Australian public surveyed was able to correctly label PTSD when presented with such a clinical vignette. By comparison, less than half that number; 14.2% of our sample was able to label the problem described in the vignette as being PTSD. This figure is even more concerning given that close to one third of participants in the current study had clinically significant symptoms that indicated a probable diagnosis of PTSD. In May et al.’s recent study [18], 84.4% of participants reported that the PTSD vignette described a mental health problem. However, recognition of PTSD *per se* was not examined in this latter study. While it could be argued that knowledge of the Western psychiatric label of PTSD is not necessary for help-seeking, there is evidence that self-labelling as having a particular mental disorder activates a schema about appropriate action to take and helps recognition of the disorder by health practitioners [15].

In terms of interventions for the problem described in the vignette, it is interesting that reading religious texts and seeking help from a psychiatrist were the interventions most often rated as helpful, given that these interventions could be seen to be at odds with each other. Indeed, one can be seen to represent beliefs regarding external higher powers while the other stems from a medical based model of mental health treatment dominant in western nations. A contentious issue in transcultural psychiatry has been the notion of whether the Western-based medical model is able to meet the needs of refugee populations, given the different cultural and social circumstances, and different views about mental health and its treatment of the latter [25]. May and colleagues [18] similarly found evidence for complex beliefs systems regarding treatment where both mental health professionals and religious leaders were considered helpful. These results suggesting the existence of complex multi-layered mental health beliefs amongst resettled Iraqi refugees are supported by our study, and have implications for treatment programs. Specifically, the study suggests that transcultural mental health services in Australia should consider ways to collaborate with traditional and religious leaders of Iraqi communities in order to better service the needs of such populations.

Of concern, was the finding that a high level of participants who believed that dealing with the problem described in the vignette alone would be helpful (58%). This was almost 10 times greater than the number of participants (6%) in the 2011 NSMHLS who endorsed dealing with the problem alone as helpful, and indicates an immediate area for improving the mental health education of refugees. Previous research on factors associated with dealing with mental health problems alone have demonstrated associations with the male gender and beliefs the disorder described is self-limiting and due to personal weakness [26]. Further research seeking to examine such associations in refugee samples is needed and will be reported upon in due course.

Other findings from our study offer more specific directions for mental health promotion and interventions. The finding that length of resettlement time influences problem recognition and beliefs in treatment helpfulness is important. For example, the finding that recently arrived respondents were more likely to believe the problem described in the vignette is ‘just a phase’ bears direct relevance to treatment seeking. Furthermore, the belief that drinking alcohol is possibly helpful held by the same sub-group is of concern and should be addressed. One way these differing beliefs can be addressed is by providing culturally adapted, translated educational material on common symptoms reported after experiencing a traumatic events, self-help strategies and treatment pathways, as part of refugee’s orientation on arrival to Australia. In terms of mental health clinicians working with resettled Iraqi refugees, it may be useful to bear in mind the possible differences in the acceptability of Cognitive Behavioural Therapy (CBT) amongst different religions. Defined in our survey as “psychotherapy focused on changing thoughts”, those from a Christian background were more likely to select it as helpful compared with those from Mandaean backgrounds. This important to note, given that CBT is considered best practice in the treatment of PTSD [27] and the widespread use amongst Western-based clinicians.

Limitations of the current study should be noted. These include the use of self-report measures of mental health and the inclusion of a measure of general psychological distress rather than separate measures of anxiety and affective disorders. Although we recruited participants from western Sydney, which has the highest concentration of resettling Iraqi refugees in Australia, given our sample were volunteers from those who attended English classes, our participants may be more acculturated than others. Strengths of the study, in our view, were the use of a culturally adapted vignette describing PTSD, the relatively large sample size, which permitted analysis of associations between demographic characteristics and measures of mental health literacy, the administration of the survey instrument by means of in-person interviews and the recruitment of participants from a non-clinical setting.

## Conclusions

In summary, the current findings suggest that resettled Iraqi refugees in Australia have low levels of problem recognition with regards to PTSD and complex beliefs systems regarding treatment. There was a numerous associations between length of time resettled in Australia, problem recognition and beliefs regarding treatment preferences, indicating a need for specific mental health education targeted at newly arriving refugees. Finally, our results suggest the need for future mental health services to consider ways in which attempts to bridge the gap between Western mental health services and traditional cultural and religious practices, thereby allowing provision of culturally acceptable and empirically based care.

## Additional file

Additional file 1:
**The vignette used in the mental health literacy survey.**

## Abbreviations

AMEP: Adult Migrant English Program
HTQ: The Harvard Trauma Questionnaire
K10: Kessler Psychological Distress Scale
MHL: Mental health literacy
NSMHWB: Australian National Survey of Mental Health and Wellbeing
NSMHLS: National Survey of Mental Health Literacy and Stigma
PTE: Potentially traumatic event
PTSD: Posttraumatic stress disorder

## References

[1] United Nations High Commissioner for Refugees (2007). Convention and Protocol Relating to the Status of Refugees.

[2] Refugee Council of Australia (2011). Australia’s Refugee and Hummanitarian Program 2012-13 Community Views on Current Challenges and Future Directions.

[3] Lischer SK (2008). Security and displacement in Iraq: responding to the forced migration crisis. Int Secur.

[4] Cronin AA, Shrestha D, Cornier N, Abdalla F, Ezard N, Aramburu C (2008). A review of water and sanitation provision in refugee camps in association with selected health and nutrition indicators – the need for integrated service provision. J Water Health.

[5] Phillips CB, Benson J (2007). Better primary health care for refugees: catch up immunization. Aust Fam Physician.

[6] Kirmayer LJ, Narasiah L, Munoz M, Rashid M, Ryder AG, Guzder J, Hassan G, Rousseau C, Pottie K (2001). Common mental health problems in immigrants and refugees: general approach in primary care. CMAJ.

[7] Taylor E, Yanni E, Pezzi C, Guterbock M, Rothney E, Harton E, Montour J, Elias C, Burke H (2013). Physical and mental health status of Iraqi refugees resettled in the United States. J Immigr Minor Health.

[8] Fazel M, Wheeler J, Danesh J (2005). Prevalence of serious mental disorder in 7000 refugees resettled in western countries: a systematic review. Lancet.

[9] Steel Z, Chey T, Silove D, Marnane C, Bryant RA, van Ommeren M (2009). Association of torture and other potentially traumatic events with mental health outcomes among populations exposed to mass conflict and displacement: a systematic review and meta-analysis. JAMA.

[10] Correa-Velez I, Sundararajan V, Brown K, Gifford SM (2007). Hospital utilisation among people born in refugee-source countries: an analysis of hospital admissions, Victoria, 1998-2004. Med J Aust.

[11] Lindert J, Brahler E, Wittig U, Mielck A, Priebe S (2008). Depression, anxiety and posttraumatic stress disorders in labor migrants, asylum seekers and refugees: a systematic overview. Psychother Psychosom Med Psychol.

[12] Gilgen D, Maeusezahl D, Salis Gross C, Battegay E, Flubacher P, Tanner M, Weiss MG, Hatz C (2005). Impact of migration on illness experience and help-seeking strategies of patients from Turkey and Bosnia in primary health care in Basel. Health Place.

[13] Morton B (2005). The health of immigrants and refugees in Canada. Can J Public Health.

[14] Jorm AF, Korten A, Jacomb P, Christensen H, Rodgers B, Pollitt P (1997). “Mental health literacy”: a survey of the public’s ability to recognise mental disorders and their beliefs about the effectiveness of treatment. Med J Aust.

[15] Jorm AF (2012). Mental health literacy: empowering the community to take action for better mental health. Am Psychol.

[16] Reavley N, Jorm AF (2011). National Survey of Mental Health Literacy and Stigma.

[17] Australian Government Department of Immigration and Border Protection (2013). Australia’s Offshore Humanitarian Program: 2012–13.

[18] May S, Rapee RM, Coello M, Momartin S, Aroche J (2013). Mental health literacy among refugee communities: differences between the Australian lay public and the Iraqi and Sudanese refugee communities. Soc Psychiatry Psychiatr Epidemiol.

[19] Bracken BA, Barona A (1991). State of the Art procedures for translating, validating and using psychoeducational tests in cross-cultural assessment. Sch Psychol Int.

[20] Kessler RC, Andrews G, Colpe LJ, Hiripi E, Mroczek DK, Normand SLT, Walters EE, Zaslavsky AM (2002). Short screening scales to monitor population prevalences and trends in non-specific psychological distress. Psychol Med.

[21] Sulaiman-Hill CM, Thompson SC (2010). Selecting instruments for assessing psychological wellbeing in Afghan and Kurdish refugee groups. BMC Res Notes.

[22] Mollica RF, Caspi-Yavin Y, Bollini P, Truong T, Tor S, Lavelle J (1992). The Harvard Trauma Questionnaire: validating a cross-cultural instrument for measuring torture, trauma, and posttraumatic stress disorder in Indochinese refugees. J Nerv Ment Dis.

[23] Dunn O (1964). Multiple comparisons using rank sums. Technometrics.

[24] Australia Bureau of Statistics (2007). National Survey of Mental Health and Wellbeing.

[25] Tribe R (2002). Mental health of refugees and asylum-seekers. Adv Psychiatr Treat.

[26] Jorm AF, Kelly CM, Wright A, Parslow RA, Harris MG, McGorry PD (2006). Belief in dealing with depression alone: results from community surveys of adolescents and adults. J Affect Disord.

[27] Association AP (2004). Practice guideline for the treatment of patients with acute stress disorder and posttraumatic stress disorder. Am J Psychiatry.

