# Supplementary material for: Mental health literacy of resettled Iraqi refugees in Australia: knowledge about posttraumatic stress disorder and beliefs about helpfulness of interventions
Source: BMC Psychiatry. 2014 Nov 18;14:320. doi: 10.1186/s12888-014-0320-x (PMC4240884; doi:10.1186/s12888-014-0320-x)
Supplement: Additional file 1: — The vignette used in the mental health literacy survey. [file 12888_2014_320_MOESM1_ESM.doc]

**Additional files**

Additional file 1 – **The vignette used in the mental health literacy survey**

Miriam* is a 37 year old married woman with 3 children, a daughter aged 7, and two sons, aged 5 and 3. Miriam has been living in Australia for the past year and has attended her local GP on several occasions with the primary complaint of an inability to sleep. The problem with her sleep started just before she left her homeland, Iraq, four years ago. Prior to leaving Iraq, Miriam was kidnapped by insurgents and held captive until her husband paid the demanded ransom. During her kidnapping, Miriam was beaten and threatened with death. She reports that during the kidnapping she felt intense fear and helpless. She reports constant nightmares in which images of death, killing and being kidnapped by masked men disturb her sleep. She avoids talking about her attack and watching Iraqi news channels in case there is a story about people being kidnapped and murdered. She is easily startled when she hears loud sounds such as a car backfiring or fireworks. She has very little interest in things around her, including her children's lives and feels little affection towards them. Finally, when questioned on how she views her future and plans for her life, Miriam replies that she does not have a future and doesn't believe she will live a long life.

*Replaced with Dawood in the case of male participants
